# A Simplified Approach to Post-Transcatheter Aortic Valve Replacement (TAVR) Conduction Disturbance Management: Bridging Efficiency and Safety in Contemporary TAVR Care

**DOI:** 10.1016/j.shj.2026.100832

**Published:** 2026-03-10

**Authors:** Jorge Nuche, Celia Denche

**Affiliations:** a Cardiology Department, Hospital Universitario 12 de Octubre, Instituto de Investigación Sanitaria del Hospital 12 de Octubre (imas12), Madrid, Spain; b Centro de Investigaciones Biomédicas en Red de Enfermedades CardioVasculares (CIBERCV), Spain

Transcatheter aortic valve replacement (TAVR) has undergone remarkable refinement over the past decade, yet conduction disturbances (CDs) remain one of its most persistent and clinically consequential complications. Despite improvements in imaging-based preprocedural planning, implantation techniques, and device design, only a minority of contemporary series report permanent pacemaker implantation (PPI) rates in the single digits.^1^^,^^2^ This is not trivial, as PPI has been consistently associated with worse long-term outcomes, including higher mortality and heart failure hospitalizations.^2^ As TAVR is increasingly offered to younger patients with longer life expectancy, the long-term consequences of TAVR-related complications become even more relevant. Supported by the rapid postprocedural recovery typical of transfemoral interventions, early discharge is strongly encouraged after TAVR. However, this benefit is often limited by the development of CDs that require extended monitoring and delay safe discharge. In this context, there is a pressing need to develop and validate simplified, pragmatic protocols that reduce PPI without prolonging hospital stay or compromising patient safety to ensure that efficiency and safety advance in parallel.

In this issue of *Structural Heart*, Faizi et al. prospectively validate a novel algorithm for the indication of a temporary pacemaker (TPM) during TAVR and for the management of CDs. The proposed algorithm limits TPM use to patients with prior right bundle branch block (RBBB) or a PQ interval > 250 ms. After TAVR, patients are subclassified into 6 subgroups based on pre-TAVR RBBB and immediate postprocedural electrocardiogram (ECG) changes and subsequently assigned to one of three discharge pathways: next-day, second-day, or third-day discharge.^3^

TPM was required in 4.5% of patients, the post-TAVR PPI rate was 11.3%, and the algorithm correctly predicted planned or early discharge in 69.8% of cases. Delayed discharge showed a distinct pattern across groups: in the next-day discharge cohort, delays were predominantly driven by non–conduction-related issues, whereas in the third-day discharge group, one-third of delays were attributable to PPI. A total of 12 patients (2%) were readmitted for PPI within 30 days, including seven with high-degree atrioventricular block. Importantly, one patient died as a consequence of complete heart block that developed after discharge; this patient did not undergo ambulatory ECG monitoring (AECG) as recommended by the algorithm.^3^

The authors should be commended for their work and for the detailed presentation of the results, which also allows identification of remaining knowledge gaps warranting further investigation.

The TPM utilization rate of 4.5% contrasts with the higher rates expected under previous management strategies.^4^ However, updated recommendations have progressively restricted the use of temporary pacing to patients with baseline RBBB; therefore, a similar or even lower rate would have been anticipated.^2^ TPM presents several drawbacks, including the need for patient immobilization and intensive care unit monitoring. The use of temporary pacing alternatives that allow patient ambulation may help mitigate these limitations.^5^

The PPI rate was 11.3%, increasing to 15.6% in the prospective evaluation of the previous expert panel validation.^6^ However, when prophylactic PPI before TAVR (indicated in patients with both RBBB and left anterior hemiblock) and acute PPI during TAVR (implanted at the treating physician’s discretion) were included, the PPI rate reached 16.1%.^3^ Prophylactic pre-TAVR PPI in patients with RBBB has been proposed in prior studies and has consistently shown a shorter length of stay without significant differences in major events.^7^ Despite its attractiveness, this strategy results in a non-negligible number of nonindicated PPIs. In the present study, nine of the 16 patients who underwent prophylactic PPI did not exhibit any ECG changes during admission, and seven of them had a ventricular pacing burden < 5% at the first outpatient visit. Therefore, refining strategies to identify which patients with RBBB are at higher risk of CD and better evaluating the need for prophylactic PPI are necessary. Pre-TAVR AECG in patients with RBBB or first-degree atrioventricular block could help detect those who already meet formal indications for PPI before the intervention.^8^ Interestingly, among the 20 patients who underwent acute PPI during the procedure, 13 had pre-existing RBBB, although prophylactic implantation had not been considered. Furthermore, the rate of right ventricular pacing in these patients was considerably higher than in those undergoing prophylactic PPI. This underscores the current inability to reliably identify which patients may benefit from prophylactic PPI before TAVR.

The proposed protocol recommends post-TAVR PPI in patients with persistent “extreme” prolongation of the PQ or QRS intervals (> 240 ms and > 150 ms, respectively) with some exceptions in which AECG is recommended. As with pre-TAVR prophylactic PPI in RBBB, this indication aims to reduce the length of stay without compromising patient safety. However, prospective validation of the aforementioned expert panel showed that patients receiving PPIs for persistent new-onset left bundle branch block (LBBB) with a PQ > 250 ms or QRS > 150 ms had a low pacing burden (2%), and ∼40% of them presented with pacing at rates below 1%.^9^ Current guidelines recommend an electrophysiological study or AECG in patients with new-onset LBBB with a PR > 240 ms or QRS > 150 ms without further prolongation for > 48 hours after TAVR and in patients with pre-existing conduction abnormalities who develop further PR or QRS prolongation > 20 ms.^2^ Interestingly, in the cited validation study, PPI based on electrophysiological study findings was not associated with a higher pacing rate.^9^ Notably, a low pacing percentage does not necessarily imply the absence of intermittent high-grade CDs that could compromise patient safety.

The low rate of sudden cardiac death or arrhythmia-related mortality confirms the good sensitivity of current protocols to identify patients at higher risk of life-threatening CDs. However, the Achilles’ heel of these protocols remains the ability to identify patients with new-onset CDs who do not require PPIs and can be safely discharged within a reasonable timeframe. This is particularly challenging in patients with new-onset LBBB. In this study, AECG was indicated in 41 patients, of whom 21 underwent AECG at the physician's discretion (out of protocol), and PPI was performed in four of them due to AECG findings. Conversely, among the 20 patients who received AECG based on protocol recommendations, only two required postdischarge PPI. Unfortunately, one patient who did not receive AECG—consistent with the protocol’s indications—died as a consequence of a severe CD. Broader use of AECG could help reduce reliance on prophylactic PPI and overcome some limitations of electrophysiological studies. However, the inability of most available devices to provide real-time monitoring prevents early diagnosis and may pose a risk for patients discharged early. The development of technologies capable of accurately classifying patients based on prosthesis-conduction system interactions or detailed analysis of intraprocedural electrocardiographic changes may help improve patient discrimination.^10^ Finally, the use of conduction system pacing or leadless pacemakers may help reduce undesirable long-term complications associated with conventional pacing strategies.

The existing knowledge gaps compel continued investigation and the development of technologies that enable early and accurate patient stratification or minimize the burden of PPI-related complications.

## Funding

The authors have no funding to report.

## Disclosure Statement

The authors report no conflict of interest.
